# 3D reconstruction of biological structures: automated procedures for alignment and reconstruction of multiple tilt series in electron tomography

**DOI:** 10.1186/s40679-016-0021-2

**Published:** 2016-06-28

**Authors:** Sébastien Phan, Daniela Boassa, Phuong Nguyen, Xiaohua Wan, Jason Lanman, Albert Lawrence, Mark H. Ellisman

**Affiliations:** 1 National Center For Microscopy and Imaging Research, Center for Research in Biological Systems, University of California, 9500 Gilman Drive La Jolla, San Diego, CA 92093-0608, USA; 2 Departments of Neurosciences and Bioengineering, University of California, 9500 Gilman Drive La Jolla, San Diego, CA 92093-0608, USA; 3 Department of Biological Sciences, Purdue University, 915 W. State Street, West Lafayette, IN 47907-2054 USA

**Keywords:** Electron tomography, 3D reconstruction, TxBR, Tomogram averaging, Iterative methods

## Abstract

Transmission electron microscopy allows the collection of multiple views of specimens and their computerized three-dimensional reconstruction and analysis with electron tomography. Here we describe development of methods for automated multi-tilt data acquisition, tilt-series processing, and alignment which allow assembly of electron tomographic data from a greater number of tilt series, yielding enhanced data quality and increasing contrast associated with weakly stained structures. This scheme facilitates visualization of nanometer scale details of fine structure in volumes taken from plastic-embedded samples of biological specimens in all dimensions. As heavy metal-contrasted plastic-embedded samples are less sensitive to the overall dose rather than the electron dose rate, an optimal resampling of the reconstruction space can be achieved by accumulating lower dose electron micrographs of the same area over a wider range of specimen orientations. The computerized multiple tilt series collection scheme is implemented together with automated advanced procedures making collection, image alignment, and processing of multi-tilt tomography data a seamless process. We demonstrate high-quality reconstructions from samples of well-described biological structures. These include the giant Mimivirus and clathrin-coated vesicles, imaged in situ in their normal intracellular contexts. Examples are provided from samples of cultured cells prepared by high-pressure freezing and freeze-substitution as well as by chemical fixation before epoxy resin embedding.

## Background

High-resolution localization and visualization of proteins and assemblies of macromolecular complexes in three dimensions (3D) within cells and tissues remain a difficult goal to achieve. With the advent of many new genetic probe-based or click chemistry-based correlated light and electron microscopy (CLEM) labeling strategies [1–3], it is desirable to facilitate production of higher quality 3D electron microscopic tomography (EMT) data sets from plastic-embedded specimens [4, 5]. In contrast to fluorescent label-based light microscopy, which provides a more limited view of the distribution of only the fluorescently labeled constituents, EMT allows the simultaneous visualization of all the intricate complexity of biological structures around the cellular or subcellular domain under investigation. However, compared to procedures for high-resolution light microscopy, EMT remains a relatively complicated method. For example, to image tissues or thicker regions of epoxy-embedded cells, semi-thin sections must generally be produced by ultramicrotomy and then imaged from many different sample orientations, yielding a set of digital electron micrographs. These micrographs must be processed through several steps to deliver the 3D volume for further analysis.

Aside from the imperfection in the optical performance of transmission electron microscopes (TEMs), such as optical aberrations [6, 7], the quality of an electron tomogram depends on additional factors. These arise from the interaction between a sample and the electron beam, the data acquisition scheme and the reconstruction process. While the electron beam damage in a plastic-embedded material mostly depends on the dose rate and not the accumulated dose [8], the overall quality of EMT data would clearly be improved from collecting more micrographs of a sample, if the micrographs alignment could be effectively optimized to handle the sample warping and other optical distortions.

In order to improve the electron tomogram quality of dose tolerant specimens, we developed a multiple-tilt series data acquisition scheme for the EMT. In contrast to the traditional single/double tilt series acquisition protocol [9, 10], this approach involves recording of a very large number of low dose or low-dose rate micrographs distributed across a wider range of specimen rotations. By sampling the specimen of interest through more orientations, the data folding into the reconstructed volume become more evenly distributed, further reducing sampling artifacts and producing higher quality tomograms. The visibility of weakly contrasted structures and thus overall specimen contrast is also enhanced by the additional summing of feature densities which accrues from the convergence of the greater multiplicity of views of the same weakly stained objects. To reduce the additional complexity of this extreme sampling and multiple tilt series reconstruction strategy, we have developed automated procedures for alignment and volume generation processes, reducing (or eliminating) the requirement for user input.

We demonstrate the utility of this method using two different test cases: the giant amoeba-infecting DNA virus Mimivirus [11–13] and a clathrin-coated vesicle in a human embryonic kidney HEK293T cell in culture. For each example, we employed both filtered back-projection and non-linear iterative reconstruction routines to generate the 3D EMT volumes. Mimivirus and the clathrin lattice assembly have been extensively studied by other methods, establishing details of their structure and thus providing useful reference data with which to compare the quality of the results obtained using our multi-tilt EMT approach.

## Methods

### Specimen preparation

Two types of specimens were used to develop and evaluate our multiple tilt EMT methodology.

To reduce preservation-associated artifacts, we examined samples of Mimivirus-infected amoebae preserved by rapid freezing, vitrified directly from the living state and processed by freeze-substitution to epoxy resin embedding. Since the structure of this virus has been studied in detail by cryoEM methods, following extraction from cells and vitrification [11, 12], we reasoned that these published findings would provide us with useful reference data to facilitate assessment of the quality of data from the multi-tilt strategy applied to stained and plastic-embedded samples described here.

Similarly, to assess our method on a well-studied ubiquitous intracellular structure following more typical mixed aldehyde fixation and plastic embedding, we examined clathrin-coated vesicles in a mammalian cell in culture (HEK293T cells). These cells were grown at 37 °C, and 10 % CO_2_ in Dulbecco’s Modified Eagles Medium (DMEM) containing 10 % fetal bovine serum (Invitrogen). Adherent HEK293T cells were grown on poly-d-lysine-coated glass bottom dishes (MatTek Corporation, MA, USA), and fixed for 30 min in 2 % glutaraldehyde in 0.1M sodium cacodylate buffer (pH 7.4) on ice for EM processing. After washes in cacodylate buffer, cells were post-fixed with 1 % osmium tetraoxide for 30 min, stained with 0.2 % tannic acid and 2 % uranyl acetate, and then dehydrated and embedded in Durcupan epoxy resin.

Sections from both sample types were cut using a diamond knife (Diatome) at a thickness between 200 and 300 nm. To improve stability of specimens under the beam of the EM, these sections were coated with carbon on both sides. Colloidal gold particles (5 and 10 nm diameter) were deposited on each side of the sections to serve as fiducial markers.

### Data acquisition protocol

EM data were obtained using an FEI Titan high base microscope operated at 300 kV; micrographs were produced using a 4k × 4k Gatan CCD camera (US4000). Both microscope and detector were controlled by the SerialEM software package [14] which managed the automated tilt series acquisition.

With recent advances in instrument automation, collection of a large number of tilt series is straightforward, and parameters to acquire all images with a relatively low-dose rate were used for this project.

The n-fold tilt series scheme we have refined and applied is a straightforward generalization of the popular double tilt series that can provide useful improvement to information in tomograms when compared to more standard single tilt series [9, 10]. In a double tilt series acquisition, the specimen is rotated $\sim$90° to obtain a combination of two tilt series, one orthogonal to the other. This is most effective in reducing reconstruction artifacts arising from the “missing wedge” problem. This two tilt series acquisition is easily achieved with a manual rotation holder such as the one offered by Fischione Instruments (Model 2040) and used in this study.

**Fig. 1:**
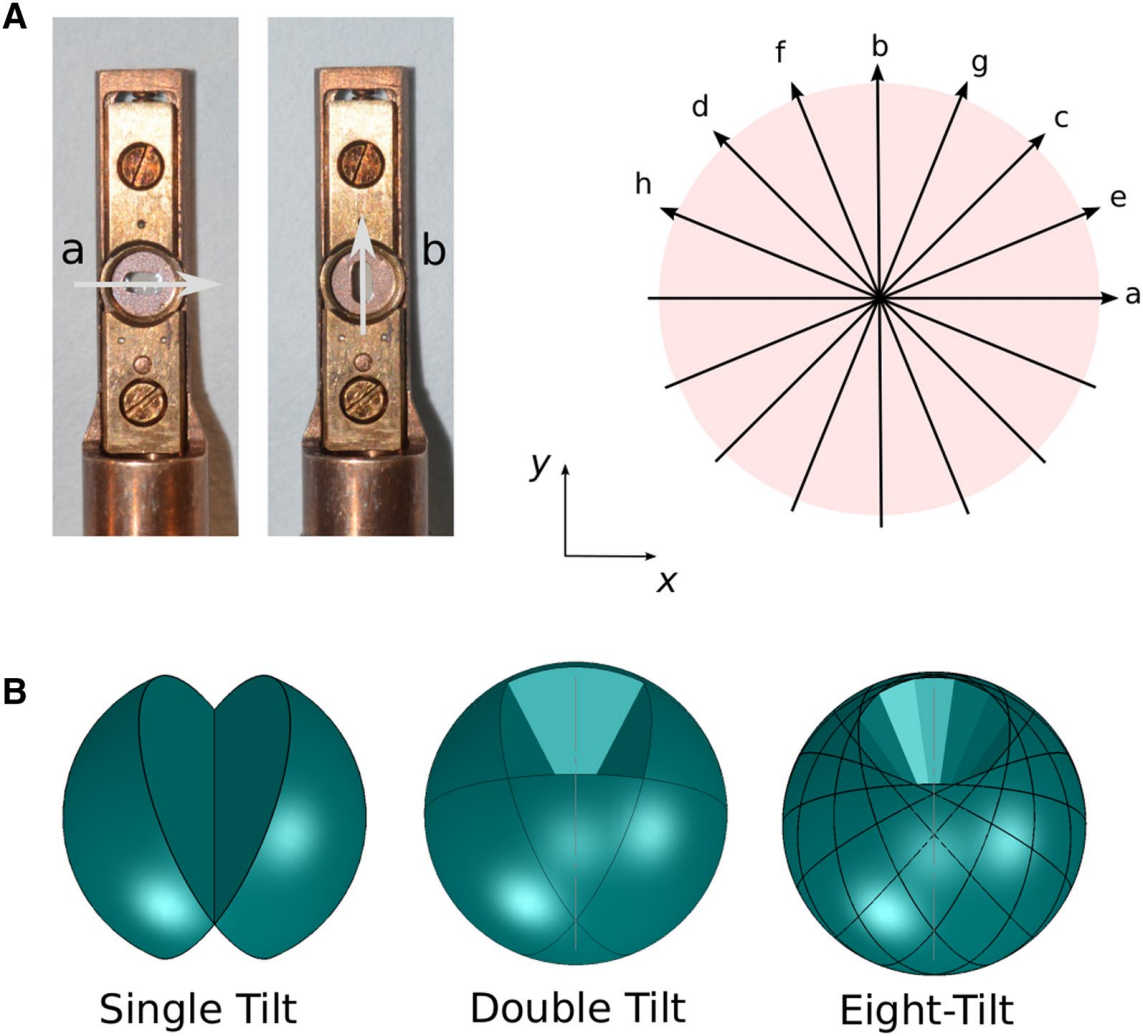
Protocol for acquiring multiple tilt series. **A** Between each series, the sample is rotated within the goniometer holder (*left side*) following an orientational multilevel access scheme (MAS) that optimizes the reconstruction space sampling. The tilt axis is displayed schematically for the 8-tilt series case (*right side*); series are labeled with letters from ‘a’ to ‘h’. The first and last series ‘a’ and ‘h’ correspond to a 0°–157.5° sample orientation, respectively.** B** 3D illustration representing the increase in accessible volume information as the number of tilt series augments

As shown below, this strategy can be extended to add additional information to the reconstruction from just two series with orthogonal tilt axes. As shown in Fig. 1, we carried out azimuthal rotations for 8-tilt series acquisitions. The specimen angles (represented by letters in Fig. 1) are arranged according to a sequence similar to the multilevel access scheme (MAS) described in [15]. By using a multilevel access scheme, the artifacts would in principle be attenuated more rapidly than alternative orders of azimuthal rotations as the number of tilt series added to the reconstruction increases.

Series up to 16-fold rotation axis and 1° tilt increments between −60° and +60° were acquired for this study; in this extreme scenario (with around 2000 projection views), the data acquisition step was completed within a single day. Each micrograph can be indexed by $i_\omega$ following the order of acquisition process (here $1 \le i_\omega \le 16\times 121$).

For the work described here, all but the specimen rotation step was automated using the serial EM scripting tools. However, full automation of such a multi-tilt strategy would only require adding in a sample holder with motorized control (and readout) for rotation. The only additional step between tilt series is to determine and adjust the stage position for the specimen to be at its eucentric height for each tilt series. Routines for automating this eucentricity adjustment are already in place in openly available automation packages such as SerialEM [14]. Projection micrographs were binned by 2 to overcome the detector limitations.

### Reconstruction procedure

To produce a high-quality reconstruction, the projection images must be aligned accurately. In TxBR [16], a reconstruction package developed at NCMIR, this is done with a non-linear bundle adjustment scheme that optimizes the micrograph registration to the final volume with the 3D distribution of markers. This registration is parametrized by means of polynomial maps [16, 17]. TxBR is able to account for a sizable amount of sample warping, optical distortion, and non-linear trajectories [18], which makes it a good candidate to align and reconstruct multiple tilt series [17].

In TxBR, the optimization procedure handles all series simultaneously. This has proved to be more accurate and efficient [10] than aligning individual reconstructions after they are generated [19, 20]. The bundle adjustment routine minimizes a gold bead marker-based reprojection error, simultaneously calculating projection maps and marker positions in 3D. In order to accomplish this, gold markers or equivalent point-like features are required. We have developed an automatic solution for both the marker tracking and projection alignment procedure that allows easy reconstruction of any* n*-fold tilt series. In summary, our method takes advantage of (i) second-order Gaussian derivatives detectors for localizing the markers [21], (ii) a cluster analysis to identify them throughout a series and (iii) iterative procedures to refine the alignment process. Details are provided in  "Appendix 1”.

Once the alignment step is completed, micrograph densities can be backprojected in the final 3D reconstruction. We have investigated two options: a regular filtered back-projection (FBP) routine and an iterative procedure requiring more complicated steps. The first approach has been described in [16] and consists of a modified Shepp-Logan filtered back-projection scheme adapted to the case of curvilinear trajectories. Our second approach, wSIRT, is a SIRT-type iterative method with an adaptive weighting scheme along trajectory paths. Compared to regular FBP, it requires multiple iterations of projection/back-projection sequences, but yields better results. It is a modified version of the so called ASART procedure introduced in [22] and recently extended to accomodate non-linear alignment [23]. In the case of incomplete phantom data, ASART was proved to generate high-quality reconstructions in comparison to other approaches [22]. In fact, the adaptive relaxation parameter used in ASART to accelerate the convergence process is responsible for attenuating some missing data artifacts.

This method can be regarded as an iterative version of cross-validation and as such can be assigned a physical meaning in the context of cross-validation [10]. It is related statistically to multiplicative-type algorithm which maximizes expectation in emission tomography [24]. A more comprehensive discussion is available in "Appendix 3."

## Results

### Sampling refinement

Traditionally in EMT, the data acquisition scheme consists in single or double tilt series [9, 10], with rather large angle increments (≃2°) in order to reduce the specimen full exposure to the electron beam radiations. In particular, assuming a full pixel resolution can be achieved, we note that the basic sampling requirement arising from a 2D analysis, $\Delta \theta = (2/L)*180^{\circ }/\pi$ [25], would not be fulfilled with the latest detector size—with for instance $L=4k$ pixels, $\Delta \theta \simeq 0.03^{\circ }$. Refined sampling schemes have been developed within the context of Fourier slice theorem to optimize the resolution of the obtained reconstructions [26, 27]. Nevertheless, the reconstruction artifacts caused by (i) the missing data issue (this is the missing wedge/pyramid problem) and (ii) the discretization problem, can only be attenuated through more sampling, and in some extent by computationally intensive iterative reconstruction methods.

Although the sampling of the reconstruction space is improved with this* n*-fold tilt series protocol, there is still a missing pyramid issue. Following  [27], it is easy to show that the fraction *q* of the missing Fourier space information is given by the following expression:

1 $$\begin{aligned} q(n) = \frac{n}{6} \tan \left( \frac{\pi }{2 n} \right) \left( \frac{L_z}{L} \frac{1}{ \tan \theta _\text{max} } \right) ^2, \end{aligned}$$

where $L_z$ and *L* are, respectively, the sample thickness and its lateral dimension, while $\theta _\text{max}$ is the maximum tilt angle within a series—usually $\theta _\text{max} \simeq 60^{\circ }$. Here, Eq. (1) is only valid when n is a power of 2, $L>L_z$ and $\theta _\text{max}>45^\circ$. When the number of tilt series equals 2 or becomes infinite, q(n) coincides well with the double tilt and conical series cases, respectively [27]. It is clear from above that accumulating the number of tilt series does not bring a large decrease in the fraction of missing Fourier information; * q*(2) = 11.1 %, * q*(4) = 9.2 %, * q*(8) = 8.84 %, * q*(16) = 8.75 %, and * q*(*∞*) = 8.73 % with taking $L=L_z$ and $\theta _\text{max}=60^\circ$. In contrast, a n-tilt series scheme allows averaging data that belong to the same Fourier space region multiple times with different sampling procedures.

### The giant Mimivirus in situ

The double-stranded DNA Mimivirus, prototypic member of the Mimiviridae family, is the primary focus of our first reconstruction example. With a genome size of about 1.1 megabases, it is one of the largest known virus. When first discovered in 1992, this virus was indeed wrongly recognized as a bacterium because of its unusual dimension and its affinity to Gram-staining [28]. Its capsid envelope is an icosahedron-like structure with a vertex-to-vertex diameter about 500 nm. The capsid presents a starfish opening on one of its fivefold vertices. Additionally, the capsid envelope is surrounded by a dense layer of fibers, bringing the overall virion size within the 750 nm range. The fine structure of the capsid envelope, which has been well characterized both with atomic force microscopy and single particle analysis on virions whose fiber coatings were removed, suggests a p3 symmetry of depressions with the capsomers being arranged in a p6 symmetry [11, 12]. This level of detail has only been reported when the external layer of fibers is enzymatically digested with lysozyme and proteases, thus drastically improving the imaging conditions of an otherwise chemically modified virus. A goal of our work was to determine if we could see these structures for virus imaged in situ (and without digestion) and if so, to determine if the multiple tilt series acquisition and processing schemes help visualize features otherwise seen only in higher resolution studies of these fiber-free isolated capsids.

We applied the EMT methodology described above to generate a 3D representation of a Mimivirus virion in situ, from a semi-thick ($\sim$150 nm) plastic section of amoebae. For reference, a projection snapshot of the area under examination is displayed in figure  when the specimen has no tilt. In this example, we used a 16-tilt series scheme at a magnification of 29 k; this corresponds to a 0.32 nm pixel size in the raw electron micrographs. For each tilt series, a 1° angular increment was used from −60° to +60°.

**Fig. 2:**
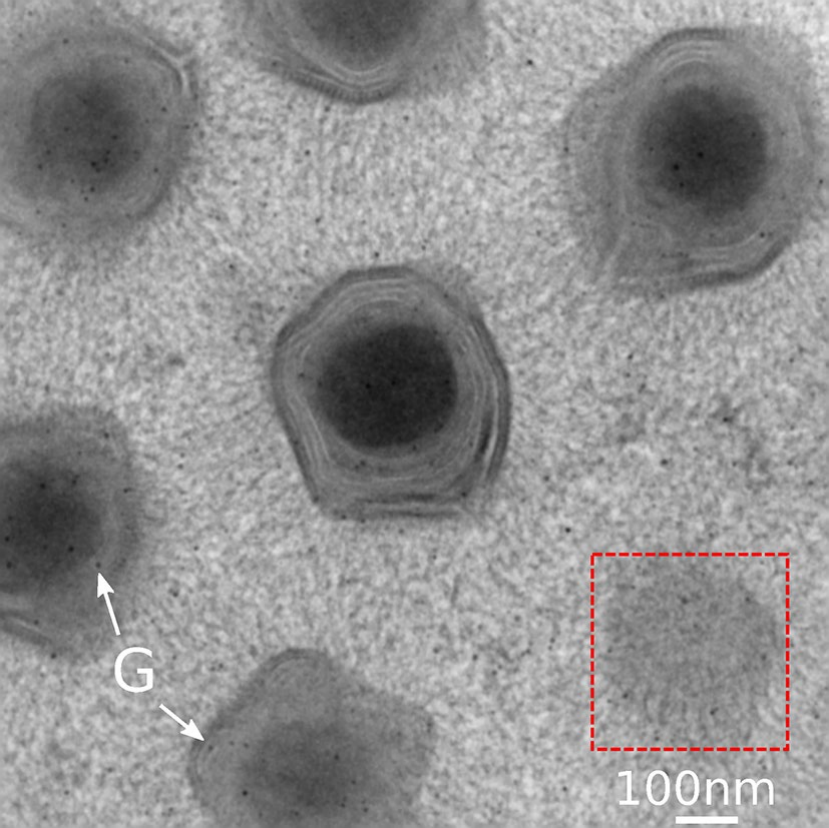
TEM micrograph of a plastic section containing Mimivirus virions infecting a cell culture at 4h post infection. The sample is at zero tilt; 5-nm gold particles (G) are used as fiducial markers for aligning the tilt series. The enclosed area corresponds to the capsid envelope portion displayed in Fig. 3

**Fig. 3:**
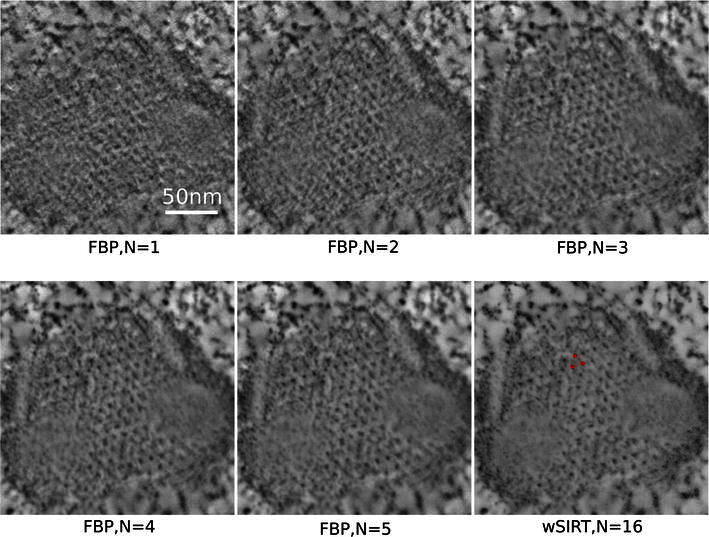
Mimivirus reconstruction: comparison of XY views of the capsid layer. Tomogram slices corresponding to the* boxed area* in Fig. 2 showing the progression in the reconstruction refinement as more tilt series (*N* = 1, 2, 3, 4, 5, 16) are included in the process, both with FBP and wSIRT. A p3 symmetry pattern of high density material in the capsid envelope (pointed by a* red triangle* in the* bottom right image*) becomes apparent, with a ${\sim}14\,\text{nm}$ peak to peak distance

Tomogram slices close to a Mimivirus capsid layer (corresponding to the boxed area in Fig. 2) built from specified numbers N of tilt series are compared in Fig. 3. Five images generated through FBP after having binned the original projection data by a factor of 2 are shown; the lower rightmost image corresponds to the wSIRT method on the* N* = 16 tilt series case. A gradual improvement with* N* is noticeable on this front view, which is theoretically the least impaired with missing information. As expected, the signal-to-noise ratio improvement is the most striking during the transition from single to double tilt, when the reconstruction artifacts are strongly reduced. Note that a p3 symmetry pattern of dense material (dark spots on the images) is clearly apparent, and consistent with the arrangement of depressions visualized in the previous studies [11, 12]. This stained material is likely to match the root of the external Mimivirus fibers connecting into the capsid depressions.

**Fig. 4:**
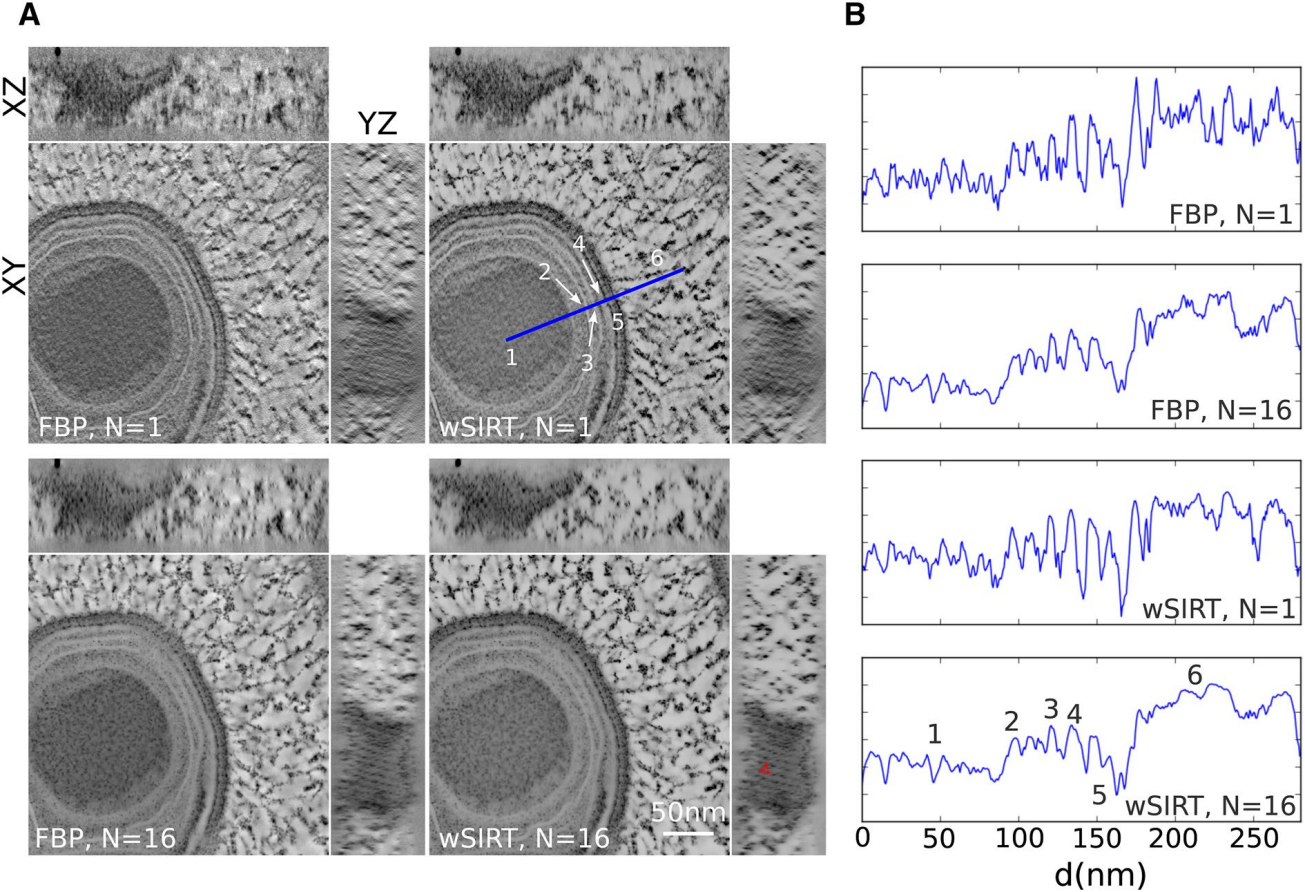
Mimivirus reconstruction obtained from different schemes.** A** Tomogram views (XY, YZ, and XZ) of a giant Mimivirus generated from the first tilt series (*N* = 1) and the whole set (*N* = 16), both with FBP and wSIRT.** B** Density profile of the various layers indicated by numbers along the* blue line* drawn in** a** as discussed in [13] obtained for each reconstruction case;* 1* DNA core,* 2* inner membrane,* 3* outer membrane,* 4* inner capsid shell,* 5* outer capsid shell and* 6* fibers

In Fig. 4, the front (XY), top (XZ) and side (YZ) views of a Mimivirus reconstruction are displayed for a single tilt (*N* = 1, Fig. 4A top row) and a 16-tilt (*N* = 16, Fig. 4A bottom row) series; the uppercase letters X, Y, and Z are used to specify the coordinates of a point in the specimen, Z being in the normal direction to the specimen slab. Results are displayed using FBP (Fig. 4A left column) and the more compute-intensive wSIRT method (Fig. 4A right column). For each case, the organization of the inner membranes of a Mimivirus virion is quite visible, and consistent with details in previous reports [13]. Benefits of our finer resampling scheme in the reconstruction space (*N* = 16 versus *N* = 1) are the most apparent on the XZ and YZ views, for which the p3 symmetry pattern structure previously discussed in XY (Fig. 3) becomes recognizable for the 16-tilt series, while it is barely discernible on the side view of the single tilt series reconstruction.

**Fig. 5:**
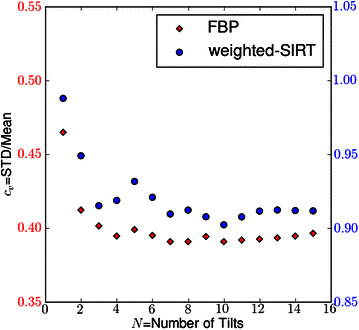
Assessment on the *convergence* process. We monitor the coefficient of variation $c_\text{v}$ on the middle tomogram slice as the number *N* of tilt series increases; $c_\text{v}$ is implemented both with FBP (*red diamond points*,* left y-axis*) and wSIRT (*blue circular points*,* right y-axis*). For the iterative approach, the total number of iterations is 50 with a mixing parameters $\alpha$ of 2; the original data were binned by 4

Density profiles along the blue line shown in Fig. 4A (top right image) were drawn for each reconstruction (Fig. 4B). It appears the signal amplitude of a single tilt (*N* = 1) reconstruction is higher compared to a 16-tilt reconstruction. This amplitude decrease with* N* should not be interpreted as a real effect: artifacts from the *missing wedge* induce a deceptive contrast into the reconstructions, with for instance white fringes around the darker features in the* XY* view (Fig. 4A, FBP *N* = 1). This explains the overall behavior of the coefficient of variation $c_\text{v}$ of a simple tomogram slice when plotted versus the number* N* of tilt series (Fig. 5); $c_\text{v}$ is defined as the ratio of the standard deviation to the mean of the pixel values.

As *N* increases, the coefficient of variation $c_v$ is first lowered (this corresponds to a decrease in the contrast), a trend only triggered by the artifact attenuation. In Fig. 5, the reconstruction process for FBP appears to converge towards a nearly stable solution, roughly after $N=8$ at which point the orientation space sampling appears fully effective.

Both FBP and wSIRT methods have been examined, and displayed a similar trend with more pronounced fluctuations for the iterative method. The total number of iteration cycles in the iterative approach was fixed to 50, and the relaxation parameter $\alpha$ was set to 2 (see Eq. (15) in "Appendix 3"). With these parameters, the relative error in the projections ended up around 5.0×10^−4^. We note the contrast enhancement between FBP and wSIRT, with clearly less white fringes around the dark features in the latter case.

**Fig. 6:**
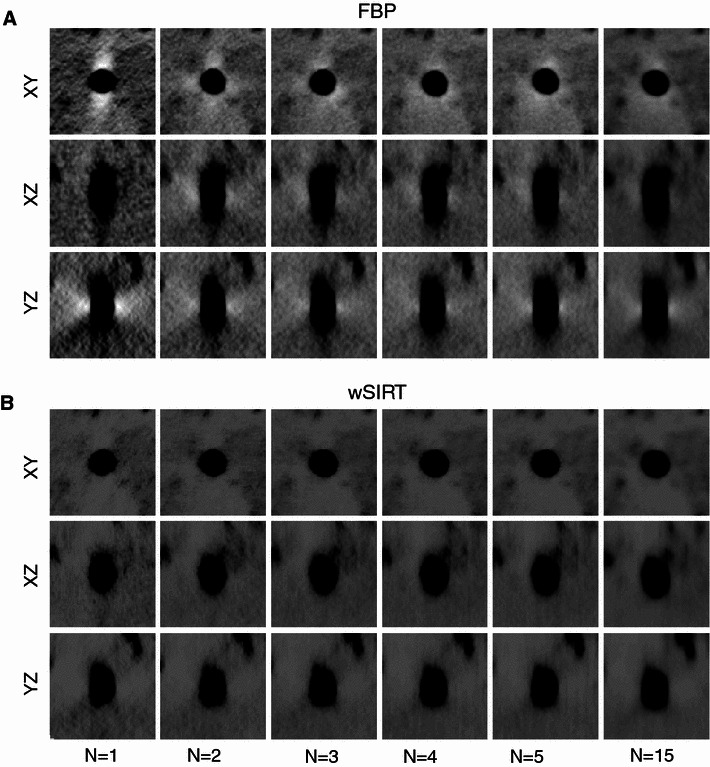
Tomogram views (*XY*, YZ, and XZ) of a 5-nm gold particle with respect to the number of tilt series N.** A** FBP is used.** B** wSIRT is used. While the image quality improves as* N* increases, the reconstruction still remain impaired by artifacts caused by the missing pyramid problem (equivalent to a missing cone problem when $N\rightarrow \infty$). Note artifacts are attenuated for wSIRT (50 iterations and $\alpha =2$) compared to FBP

For completeness, the front (XY), top (XZ), and side (YZ) views of a 5-nm gold particle are displayed in Fig. 6. Despite the improved sampling (with* N* = 1,...,15), artifacts around gold particles from the missing pyramid (or cone) are still slightly visible in any FBP reconstruction. However, they are greatly attenuated in the wSIRT reconstruction (Fig. 6).

**Fig. 7:**
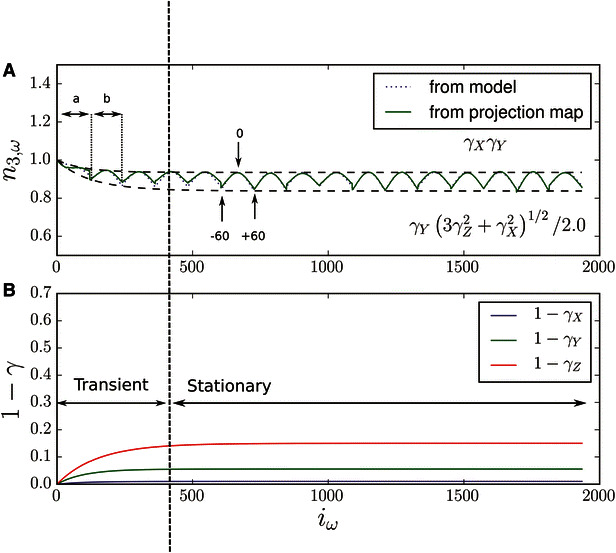
Sample warping caused by the electron beam radiation can be simultaneously monitored and corrected for in the multiple tilt process.** A** The variations of $n_{3,\omega }$ (*green lines*), defined in Eq. (10) and based on the linear portion of the computed projection maps, are a good indicator of the sample deformation happening over the course of the data acquisition. Its main behavior can be reproduced (*doted blue lines*) by considering a simple model having three deformation rates ($\gamma _X$, $\gamma _Y$ and $\gamma _Z$) allowing their estimates after an adjustment procedure. $n_{3,\omega }$ oscillates between two curves as the many different tilt series (a, b,…, p) are successively acquired between −60° and +60°. The* lower bound curve*, reached at high* tilt angles* ($|\theta | =\pi /3$), specifically depends on the sample compression along the normal axis, while the* upper bound curve*, reached at zero tilt, only depends on the lateral compressions. **B** Representation of the amount of compression along the three main sample directions versus the micrograph index. In the stationary region, the sample compression is estimated to be 2–4 % in the lateral directions and 15 % along the normal axis

The electron dose rate applied onto the Mimivirus sample over the data acquisition course was maintained at a low level, within 2–3 electrons per Angstrom square per second. However, this is enough to induce some obvious specimen deformations, as previously reported [8]. As described above, the bundle adjustment procedure used here in conjunction with a general projection model for aligning the micrographs offers an effective solution to the problem as shown in Fig. 7. After analyzing the final projections maps, we found that the corrected sample deformation is quantitatively in line with [8], showing a compression factor up to 15 % along the normal axis to the section and half or less along the lateral directions. Correction of this sample deformation is provided during the alignment process ensuring a minimal re-projection error [16–18]. To estimate the compression factors, we considered a simple deformation model with only three deformation coefficient rates along the X, Y, and Z sample directions, see "Appendix 2" for more details. With this model, we are able to capture the main variations of the final projection maps with a minimal set of adjustment parameters. As an illustration, we plot in Fig. 7 the quantity $n_{3,\omega }$ defined in Eq. (10) versus the micrograph index $i_\omega$, both for the projection maps computed from the bundle adjustment and the ones from the simple deformation model. In an ideal system with in particular no sample deformation, the quantity $n_{3,\omega }$ should remain equal to 1 by design; deviations from 1 would indicate a possible specimen warping. Once the model is adjusted, the prediction for $n_{3,\omega }$ roughly follows the computed map estimates as shown in Fig. 7, validating our choice of model. Note that the Z compression rate contribution to $n_{3,\omega }$ is larger at high tilt angles compared to its in-plane (*X* and* Y*) counterparts, explaining the oscillatory behavior of $n_{3,\omega }$ versus the micrograph index $i_\omega$. One interesting feature of this analysis is the ability to monitor both the transient and stationary sample deformation while the specimen is irradiated, the stable state being reached after 3–4 tilt series in this experiment. It is worth noting that while some EMT practices for recording data from epoxy-embedded samples include a preliminary long exposure beam “cooking” step prior to the image acquisition protocol, this can certainly be viewed as contributing to the alteration of the sample dimensions (particularly its thickness) and potentially a loss of useful information. However, as long as the transient behavior can be accurately modeled through a low-dose rate strategy and a refined tomographic alignment scheme, it is possible to ensure the final reconstruction geometry to be close to that of the original sample when it was first inserted into the TEM.

### Clathrin-coated vesicles

**Fig. 8:**
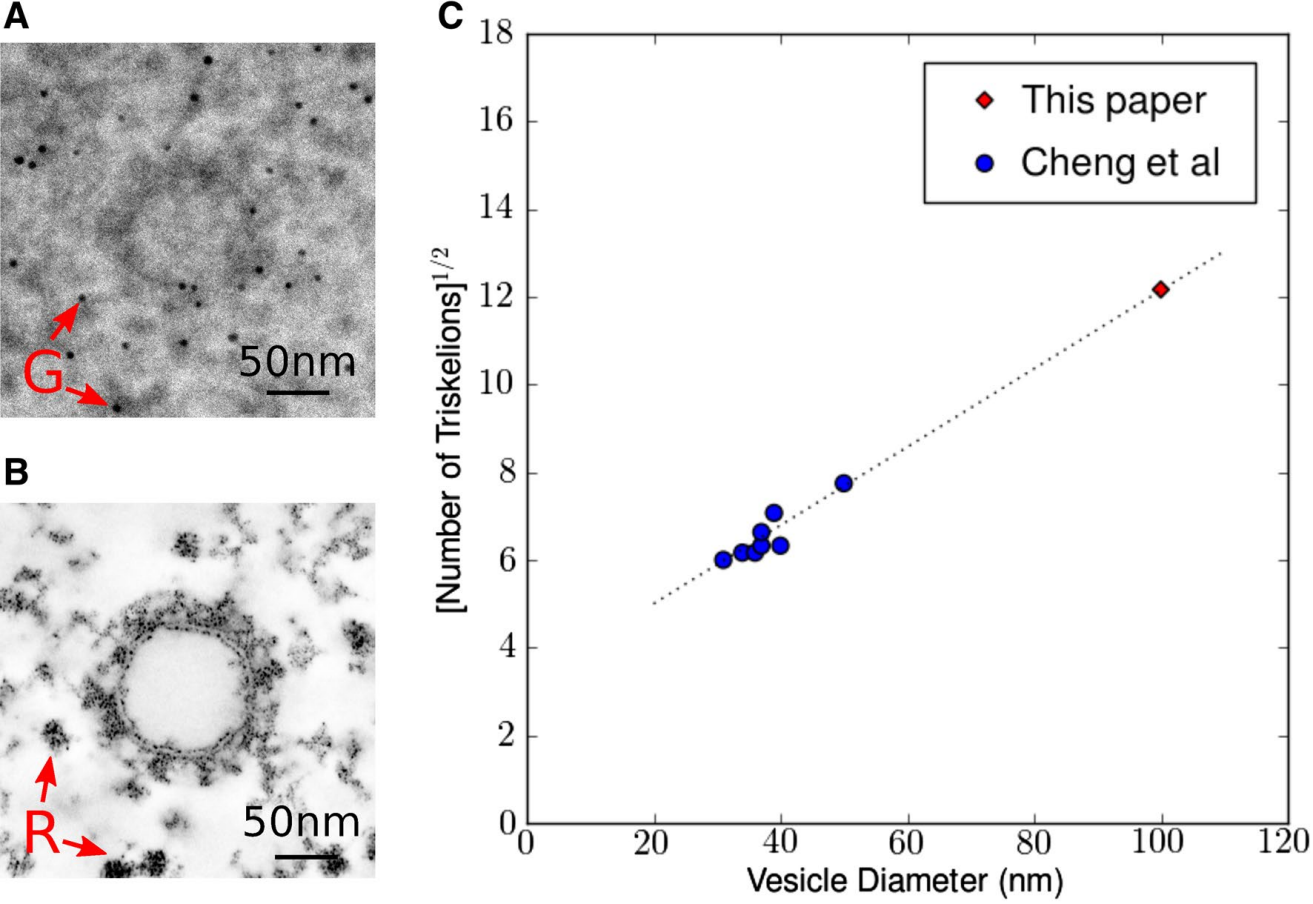
Clathrin-coated vesicle reconstruction using a 16 tilt series scheme.** A** EM snapshot at zero tilt;* gold markers* (G) are indicated with* arrows*.** B** *XY* section in the middle of the reconstruction. Note the contrast increase between the projection in A and the tomogram slice. Free ribosomal units (R) are indicated with* arrows*. The diameter of this organelle inner vesicle averaged around 100 nm, making it significantly larger than previously reported similar structures [30].** C** The number of triskelions should increase linearly with the vesicle surface; we counted 143 triskelions, which is consistent with the trend reported in [30]. Indeed, the square root of triskelions number versus inner vesicle diameter roughly follows a* straight line*.

**Fig. 9:**
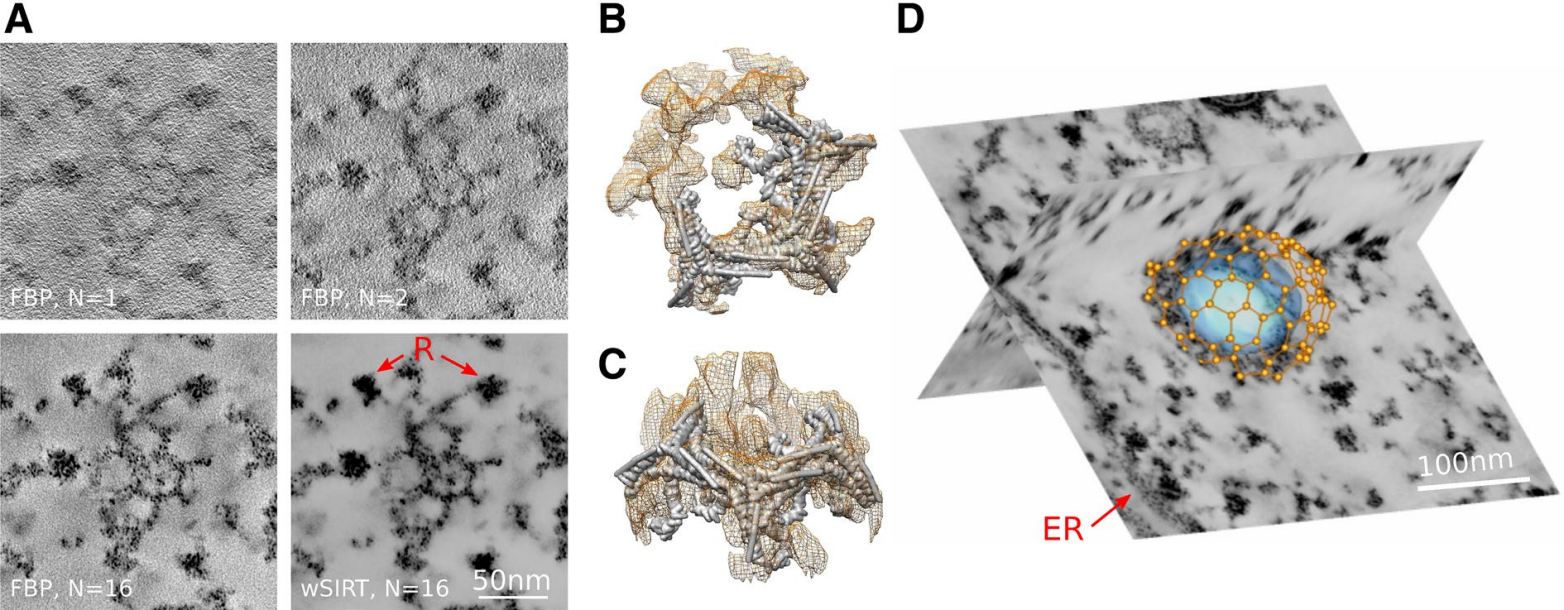
Clathrin-coated vesicle reconstruction using different schemes.** A** *XY* tomogram slices containing a partial view of the clathrin cage obtained with different reconstruction schemes (FBP,* N* = 1, 2 and 16; wSIRT, * N* = 16). Note the free ribosomal units (R) around the cage.** B**,** C** An isosurface of a portion of the cage reconstruction (*orange wireframe*) is compared to the corresponding theoretical protein model assembly (*solid gray surface*). No extra matching transformation other than simple translations/rotations is necessary.** D** A segmentation of the clathrin cage is displayed on a wider scale showing a rough endoplasmic reticulum (ER) as well

Clathrin-coated vesicles mediate the vesicular transport of cargo such as proteins between organelles in eukaryotic cells connecting the trans-Golgi network, endosomes, lysosomes, and the cell membrane. Clathrin is a large protein complex composed of three heavy chains and three light chains. This complex is referred to as a triskelion based on its three-legged appearance; it coassembles together with adaptor proteins to form the ‘coat’ around the vesicles. The spherical clathrin lattice structure appears as a polyhedron made of regular pentagons and hexagons. The adaptor proteins serve as links between the clathrin lattice and the membrane of the vesicle. The lattice structure of highly symmetrical forms of clathrin-coated vesicles assembled in vitro has already been studied in detail from single particle analysis [29]—for instance the mini-coat with its tetrahedral symmetry and the hexagonal barrel. Cryo-electron tomography reconstructions of lesser symmetrical clathrin-coated vesicles isolated from cells have also been reported [30]. We present here a sub-volume reconstruction of a cell in culture containing an individual coated vesicle (Figs. 8, 9). For this, we used a TEM magnification of 37 k and a 16 tilt-series scheme with 1° angle increment between −60° and +60°. Compared to the previously reported studies of isolated clathrin-coated vesicles, this in situ instance is a large assembly containing a 100 nm diameter inner vesicle; the outer coat diameter being measured at roughly 140 nm. Note that the overall shape is not perfectly spherical but slightly oblong. We counted 61 hexagons, 11 pentagons, and 1 heptagon on the cage surface, which includes a total of 143 triskelions and 214 edges. Those numbers are consistent with prior studies as we expect them to behave linearly with the overall vesicle surface (Fig. 8C). In Fig. 8A, a micrograph snapshot of the vesicle is displayed when the sample is not tilted; specimen features are then hardly discernible. This is not the case on a tomogram slice once the 16-tilt reconstruction is performed (Fig. 8B). The wSIRT reconstruction was generated after 50 iterations and a mixing parameter $\alpha =2$; the original data were binned by 2. The clathrin cage structure imaged here is within the functional context of the cell, a major difference from previous studies which were also carried out using cryoEM utilizing different contrast mechanisms. As this is an in situ-coated vesicle, its relationship with other cellular elements such as free ribosomal units can be seen directly, as indicated in Figs. 8B and 9A; a portion of rough endoplasmic reticulum is also visible in Fig. 9D.

In Fig. 9B, C, we matched the theoretical model of a basic molecular assembly made of three consecutive triskelions to our EMT reconstruction. For a larger scale perspective, the clathrin cage vertices, which also correspond to triskelion center locations, are displayed in Fig. 9C.

## Discussion

In this work, we established automatic procedures to facilitate the processing of EMT reconstructions. This enabled us to introduce an averaging-like strategy to increase contrast of weakly stained structures based on multiple tilt series acquisition, thereby enhancing visibility of fine structure in 3D. This strategy was applied on two distinct samples providing examples of its benefits and also demonstrating the use of two reconstruction procedures, a regular FBP and wSIRT, a weighted iterative procedure. High-quality reconstructions were obtained with both procedures.

Use of a specific variation of an EMT image acquisition and reconstruction strategy should be determined based on the requirements of the project and the characteristics of the specimen being studied.

With frozen hydrated samples, a weak signal to noise ratio and a low radiation tolerance are the key concerns. Cautions must be taken to decrease the damage from electron interaction with the objects of interest. Techniques to boost the contrast are used to enhance image information, either with a strong defocus, an imaging energy filter or a phase plate [31]; this, in return, requires additional corrections with for instance a phase flipping step [32].

In contrast, considering the case of plastic-embedded material, as the electron dose rate appears to be more limiting than total dose, more extensive data acquisitions are possible especially if the dose rate is low. This was applied in our work. Contrast and resolution of objects within the tomograms are improved by increasing the angular density of projections and processing the resulting set of images with software designed for this purpose. Efficiency and accuracy in aligning large sets of micrographs then become the critical practical challenge to overcome in 3D reconstruction. Our procedure can be viewed as a variant of averaging to improve signal to noise, combined with multiple tilt-based improvement on sampling from a greater number of perspectives.

Many other approaches in modern 3D EM rely on averaging. In single particle analysis [33], the reconstruction process involves a large collection of snapshots containing identical structures with random orientations. This technique offers the opportunity to obtain very high-resolution average-based reconstructions representing the features of small molecular assemblies. Averaging is also possible in 3D [34, 35]. Unfortunately, the quality of the products of this process can be hampered by the contributions of reconstruction artifacts which therefore need to be accounted for during the image processing.

Our approach focuses on a single specimen area within a rather large field of view, and seeks to redistribute the sample dose images taken over many orientations. It can be likened to some 3D tomogram averaging aspects, the difference being we are averaging data from exactly the same volume, the specimen only being rotated around the optical axis between each series, and it would be difficult to totally overcome the missing wedge issue.

With a large number of exposures/tilt series, marker-based registration for the EMT process remains very effective. The automatic alignment procedures developed in this work allow us to handle a high density of gold particles, with thousands of markers in the field of view. In the two examples we considered that the total number of gold particles used for alignment is 365 and 60 for the giant Mimivirus and clathrin-coated vesicle, respectively. We successfully tested our alignment routines on other reconstructions containing several thousands markers. While the presence of numerous markers might be detrimental to the reconstruction, with streak-type artifacts, it also allows for very accurate image registration especially when using polynomial map correspondences as in TxBR. Artifacts surrounding a gold particle—an electron dense material on a dim background—can be limited if its diameter is chosen small (a few pixels), or if the weighted iterative method is used (Fig. 6).

wSIRT brings significant improvements over FBP. It enables a clear contrast enhancement and a large artifact reduction (Figs. 5, 6). As a downside, this iterative approach is compute-intensive and requires hundred times more operations compared to FBP. Nevertheless, it is possible to make modifications in our current routine to accelerate the convergence process. For instance, one could gradually include the tilt series into the iterative computations, similarly to the approach we took taken in MAS data acquisition protocol, as shown in Fig. 1.

In a high throughput EMT production environment, implementing wSIRT with a very large number of tilt exposures might be difficult to carry out as a routine process. This leads to the practical dilemma of whether to acquire a small number of tilt series (typically ranging from 2 to 4) with the iterative approach, or to acquire a large number of tilt series and restrict the reconstruction process to filtered back-projections.

The answer to this question may reside in the properties of the specimen preparation itself. For a sample with a strong level of heavy metal staining, use of a large number of tilt series might not be as beneficial since the incoming signal to noise ratio can be comparatively high. More data averaging will not likely bring out the details of unstained or weakly contrasted biological features in the reconstruction, while the high-resolution features can be muddied by over-staining. In this case, implementing wSIRT on fewer tilt series may be judicious. A “multiple tilt and summed view averaging technique”—like the one presented here—may be more useful at lower magnification. For a sample with low staining level, this averaging technique would probably bring more reconstruction details than a regular tomographic process (with a single/double tilt series scheme), as our example on the clathrin-coated vesicle seems to indicate. Further investigations in this direction need to be done.

## Conclusions

We believe that the developments described above provide a useful method to enhance resolution of fine detail in tomographic reconstructions from plastic-embedded and heavy metal stained samples, thereby progressing a general goal of revealing supramolecular details in situ. While this approach addresses the under-sampling and noise averaging issues, it does not overcome the ambiguity related to a missing cone in the raw data. The impact of this missing data nevertheless remains small, especially when a weighted iterative procedure is applied.

**Fig. 10:**
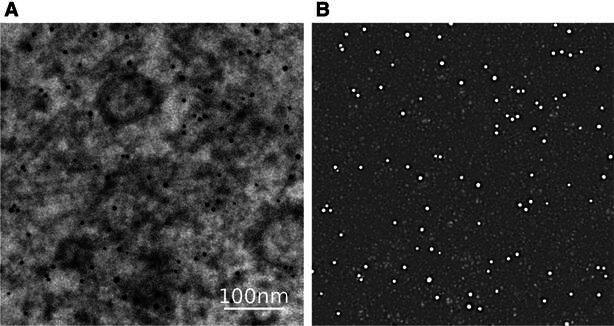
Demonstration of the* gold marker* detection scheme outlined in "Appendix: Marker detection." This filtering procedure makes use of gaussian derivatives to calculate the second-order derivatives and allows to selectively enhance markers of a specific size (see recipe [21]).** A** A projection micrograph of the clathrin specimen at zero tilt is displayed.** B** The corresponding* detector image* is shown. The biological background behind the 5-nm gold markers is largely attenuated after applying this filter.

Acquiring many micrographs at a lower dose rate, as we demonstrate in this work, could be useful in a full 180° EMT process as well. In such a setup, the biological specimen should be more sensitive to the beam-associated mass loss given its geometry, a few hundred nanometers diameter cylinder with isolated supports (Fig. 10). Micrographs in this particular application should be distributed within a single series as an unequivocal mathematical solution to the problem is available.

Finally, additional refinements to this multiple tilt series reconstruction strategy are still possible. For instance, registration adjustments can be included at each iteration step *i* of the wSIRT approach. A small misalignment in the micrographs creates systematic patterns in the projection errors $v-v_i$ [see "Appendix 3" Eq. (15)], which can be reused as a feedback to correct for the original projection maps. This would accelerate the convergence of this recursive approach, and also improves the quality of the completed reconstruction on the smaller scale.

## Figures and Tables

**Fig. 11:**
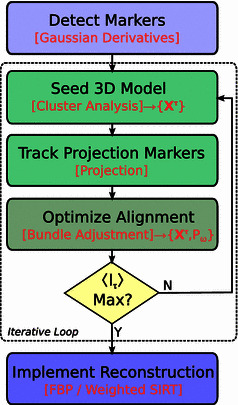
Flow chart describing the automation process for a single tilt series reconstruction. After detecting the marker projection peaks throughout the micrographs, 3D marker positions $\{\mathbf{X^\tau }\}_\tau$ are inferred using a cluster analysis; this analysis is carried out on a set of 3D points located at the *cross-over* nodes of back-projected rays (Fig. 12). A trace ($\equiv$ set of matching peaks in a micrograph series) is then estimated for each marker, and the projection maps refined with a bundle adjustment procedure. This process is repeated iteratively to maximize the average trace size $\langle l_\tau \rangle _\tau$

**Fig. 12:**
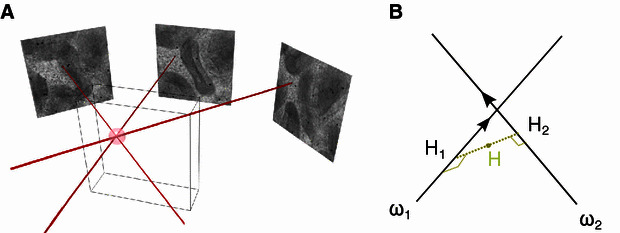
The 3D seeding procedure.** A** The* gold markers* are inferred as the main convergence nodes of back-projected rays arising from the detected projection peaks.** B** In practice, a cluster analysis is performed on the mid-points of the shortest segment joining selected pair of back-projected rays. Note that the two paths $\omega _1$ and $\omega _2$ do not necessarily cross in 3D

**Fig. 13:**
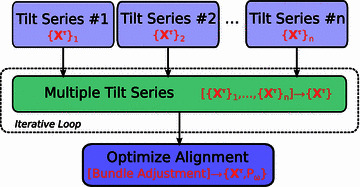
Flow chart summarizing the automation strategy for multiple tilt series. Each series *i* is first reconstructed independently as outlined in Fig. 11, generating multiple sets of gold marker 3D positions $\{ \mathbf{X_i^{\tau _i}} \}_{\tau _i}$. Their correspondences are established through an iterative process, allowing to identify the matching traces between tilt series. A global alignment is then implemented

## Appendix 1: Automatic alignment

The averaging method proposed in this work relies on automating the EMT reconstruction process for any number of tilt series. This appendix describes in detail our procedure steps: (i) the marker detection, (ii) the single tilt alignment, (iii) the multiple tilt alignment. The back-projection routine in the case of the wSIRT approach is explained in the next appendix. The general alignment flow is summarized from Figs. 10–13.

### Marker detection

The precise alignment of a tomographic series typically depends on fiducial markers; it is achieved by spreading gold particles on the specimen surfaces. By tracking the marker projections throughout a series of micrographs, the specimen deformation can be closely monitored and accounted for in the final alignment. This enables accurate electron micrograph registration, and ultimately leads to high-quality reconstructions.

The first step towards fully automated EMT consists in detecting the gold particles for each electron micrograph. We follow the procedure outlined in reference [21]. Since gold particles on a micrograph correspond to small black disks, the method consists in finding local maximum in the second-order derivatives (or equivalent) of the image gray levels. Following [21], this detection scheme is optimized to be selective for a certain length scale $\sigma$ related to the marker size. Hence, the gaussian derivative concept is introduced. Second-order gaussian derivatives with a scale level $\sigma$ are simply obtained by convoluting images with three kernels $G^{\sigma }_{xx},$ $G^{\sigma }_{xy}$ and $G^{\sigma }_{yy}$:

2 $${I^{\sigma}}_{ij}(x,y) = I(x,y) * G^{\sigma}_{ij}(x,y)$$

with *i*, *j* in $\{x,y\};$ *x* and *y* denote for the two coordinates of an image pixel.

3 $$\begin{aligned} \begin{array}{l} G^{\sigma }_{xx}(x,y) = \frac{x^2-\sigma ^2}{2 \pi \sigma ^4} \exp { \left( - \frac{x^2+y^2}{2 \sigma ^2} \right), } \\ G^{\sigma }_{yy}(x,y) = \frac{y^2-\sigma ^2}{2 \pi \sigma ^4} \exp { \left( - \frac{x^2+y^2}{2 \sigma ^2} \right) ,} \\ G^{\sigma }_{xy}(x,y) = \frac{x y}{2 \pi \sigma ^4} \exp { \left( - \frac{x^2+y^2}{2 \sigma ^2} \right). } \\ \end{array} \end{aligned}$$

The detector image is then built from the eigenvalues $\lambda _\text{min}$ and $\lambda _\text{max}$ of the 2 by 2 image matrix $\left\{ I_{ij} \right\}$. In our case, we choose to detect particles from the following quantity $D=\left| \lambda _\text{min} \lambda _\text{max} \right|$.

Peaks in image *D* are considered positive if their normalized correlation coefficient is above a certain threshold. The threshold value can be readjusted afterwards (in a second pass) so the number of selected peaks, i.e., the number of gold markers, remains consistent throughout the tilt series.

### Iterative alignment for single axis tomography

Immediately after the detection, the set of peaks is still an unclassified collection of points. It is not possible to distinguish any trace corresponding to a specific marker within the micrograph series.

This is resolved using a procedure based upon a cluster analysis to identify possible gold particles 3D locations and their associated projections. Precisely, when back-projecting rays arising from detected peaks, markers should be located on the convergence nodes (Fig. 12A). Once a marker position is assessed, its projection trace can be rebuilt by picking the most probable matching peak on each micrograph—this is the closest peak to a micrograph projection point within a given distance.

The overall automation scheme is outlined in Fig. 11. It is an iterative procedure that repeatedly builds projection traces from cluster seeds and refines the micrograph alignment until the average peak number in the traces becomes maximal. The initial state in this iterative routine corresponds to a rough alignment where micrographs are aligned by means of simple cross-correlations. It has consistently shown to lead to the correct final alignment.

In practice, the cluster analysis is carried out on a set of 3D points generated from pair of back-projected rays; for each pair of rays, the mid-point of the shortest segment joining them is retained; this would correspond for instance to mid-point *H* of segment $H_1 H_2$ in Fig. 12B. Narrow regions exhibiting a high point density are mapped to clusters; their centroid is taken as the 3D position of a marker and used as an input to determine the projection traces. Throughout this computation, the projection maps $P_{\omega }$ that associates a 3D point $\mathbf{X}$ to a 2D pixel $\mathbf{x}$ in micrograph $\omega$ are taken affine, i.e., rays are considered as straight lines. Also, to ensure the 3D seeding procedure is efficient, we do not consider all the possible pairs of rays, but only the ones separated by a fixed angle increment—for instance 20°. By doing so, aligning tilt series containing thousands of markers can be done in a matter of minutes on a regular workstation. To implement the cluster analysis, we used the **scipy.cluster** package [36].

After the seeding procedure has been initiated and the marker traces been identified, the alignment is finally optimized through a bundle adjustment procedure by minimizing a reprojection error. This allows to refine the projection maps $P_{\omega }$ ; this technique has been extensively discussed in the context of electron tomography [16–18].

When markers are numerous (for instance, more than $n_\text{c}=100$), the procedure outlined in Fig. 11 is implemented twice. At first, we restrict the number of markers to be less than a certain threshold (${<}n_\text{c}$) but still evenly distributed; this allows to quickly estimate the projection maps $P_{\omega }$. In a second pass, all the particles are considered: since the initial value of $P_{\omega }$ is already very accurate, the procedure remains efficient. A second pass might be unnecessary for single tilt series. This is not the case for multiple tilt series as we will see in the next paragraph. Also, note there is no guarantee every single particle is picked by our automation routine; it is nevertheless very effective because of the two-pass detection scheme—see Appendix "Marker detection".

Compared to other approaches, for instance the *beadtrack* utility in eTomo [20], our procedure uses a global (3D) framework; it does not try to connect peaks directly from one micrograph to the next one in a sequential manner. This is still possible to achieve with geometrical cross-over consideration, but probably not optimal: there is a large uncertainty on the cross-over location of two nearly parallel rays. Finally, we note that a sequential cross-correlation procedure is used during the built up of the initial projection map.

The seeding step depicted in Fig. 12 and its cluster analysis could be excluded from the iterative loop and implemented only once as an initial routine. Maintaining this step within the loop allows the number of markers to grow and thus more alignment flexibility, with only a limited impact on the algorithm performance.

Once the iterative routine has converged, a final bundle adjustment scheme can be implemented using non-linear projection maps [16, 17]. This would insure a more accurate alignment, and eventually correct for any morphological distortions [18]. For multiple tilt series however, this non-linear bundle adjustment should be carried out when the entire procedure is completed.

### Iterative alignment for multiple axis tomography

The automated routine described above can be applied to any number of tilt series without substantial modification, just by considering them as a unique dataset/series. A more robust methodology consists in aligning first each series separately, and handle them globally once their individual signatures—projection map $\{P_\omega \}_\omega$ and marker locations $\left\{ \mathbf{X}^{\tau } \right\} _{\tau }$—are known. This is the approach we adopt; the workflow is summarized in Fig. 13.

For the global alignment, correspondences between markers from different series need to be established. This task is implemented with another iterative procedure.

In the following, $\left\{ \mathbf{X}^{\tau _a}_{a} \right\} _{\tau _a}$ and $\left\{ P_{\omega _a} \right\} _{\omega _a}$ indicate respectively the 3D marker positions and projection maps for tilt series *a*; $\tau _a$ indexes the markers and $\omega _a$ the specimen orientation.

For every pair of series (*a*,*b*), two functions $\alpha _{ab}$ and $\alpha _{ba}$ map the correspondences between the sets $\left\{ \mathbf{X}^{\tau _a}_{a} \right\} _{\tau _a}$ and $\left\{ \mathbf{X}^{\tau _b}_{b} \right\} _{\tau _b}$:

4 $$\begin{aligned} \begin{array}{l} \tau _b = \alpha _{ab}(\tau _a) \\ \tau _a = \alpha _{ba}(\tau _b). \end{array} \end{aligned}$$

Since reconstructing from *a* or *b* is *equivalent*, we introduce $T_{ab}$ (resp. $T_{ba}$), the invertible 3D transformation to switch from volume *a* to volume *b* (resp. *b* to *a*); $T_{ab}^{-1}=T_{ba}$. In this approach, the index mapping $\alpha _{ab}$ is set by comparing the point to point distance between $\{ T_{ab} ( \mathbf{X}^{\tau _a}_{a} ) \}_{\tau _a}$ and $\{ \mathbf{X}^{\tau _b}_{b} \}_{\tau _b}$; this allows to assign a unique $\tau _b$ for each $\tau _a$ as long the corresponding distance is short enough. A similar procedure is used with $\alpha _{ba}$. At the end, only tracks $\tau _a$ from *a* satisfying the condition $\tau _a = \alpha _{ba} \circ \alpha _{ab}(\tau _a)$ are kept. Those tracks and their *b* counterpart are then used to refine the transformation $T_{ab}$ with a 3D regression procedure. This process can be repeated iteratively until the number of shared markers between the two sets is maximized.

For the first iteration, the 3D transformation $T_{ab}$ is initialized as a simple 3D rotation around the *Z* axis. Its parameters, i.e., translation coefficients and rotation angle, are estimated by comparing the *zero-tilt* images from both series. The images are cross-correlated for different relative rotations; the highest correlation peak provides necessary elements to estimate $T_{ab}$.

This iterative operation is repeated for each pair of tilt series. Once all the shared markers are identified, the original marker sets are recollected as a unique set, and each projection maps redefined in relation to the first series *a*, that is $P_{\omega _b} \rightarrow P_{\omega _b} \circ T_{ab}$. Those quantities are used as inputs in a final bundle adjustment procedure for the entire system.

It is clear that having detected a maximum number of markers during the single tilt series alignment is important to establish the geometrical correspondence between tilt series.

## Appendix 2: Monitoring the sample deformation

Part of our present knowledge about electron beam damage on plastic sections derives from studies relying on tracking fiducial markers [8]. It therefore comes as no surprise that the same information is inherently contained in any EMT process whose alignment procedure shares the same principles. In fact, the overall quality of a reconstruction technique depends on how well the alignment procedure compensates for sample deformation, among other issues.

We describe in this appendix how to simply extract some sample deformation parameters from the projection maps $\left\{ P_{\omega } \right\} _{\omega }$, once the bundle adjustment optimization procedure has been carried out.

In our procedure, the correspondence between a point $\mathbf{X}=(X,Y,Z)$ of the specimen at no tilt and a projection point $\mathbf{x}=(x,y)$ on a micrograph for a given specimen orientation $\omega$ is expressed as a polynomial expansion, which at the first-order writes:

5 $$\begin{aligned} \left\{ \begin{array}{rcl} x &{} = &{} t_{x,\omega } + n_{1X,\omega } X + n_{1Y,\omega } Y + n_{1Z,\omega } Z + ... \\ y &{} = &{} t_{y,\omega } + n_{2X,\omega } X + n_{2Y,\omega } Y + n_{2Z,\omega } Z + ... \\ \end{array} \right. , \end{aligned}$$

the linear portion of this development being characterized by a set of two vectors ${ \mathbf n}_{1,\omega }$ and ${ \mathbf n}_{2,\omega }$ at each sample tilt.

In an ideal system, with no sample warping and optics following straight line rules, the projection map in (5) would exactly correspond to projecting the result of two simple rotations: $\Pi _\mathbf{e_Z} \circ \mathcal{R}_\mathbf{{u},\theta } \circ \mathcal{R}_{\mathbf{e_Z},\phi }$; the first one $\mathcal{R}_{\mathbf{e_Z},\phi }$ modeling the sample replacement between each tilt series ($\phi$ is the azimuthal angle), while the second one $\mathcal{R}_\mathbf{{u},\theta }$ describes the sample tilt operation within a series, $\mathbf{{u}}$ is the tilt axis ($\theta$ is the tilt angle), and $\Pi _\mathbf{e_Z}$ the orthogonal projection along the optic axis $\mathbf{e_Z}$.

At a high enough magnification to avoid any optical distortion problem, and with a small electron beam dose rate, we expect the deviations from the ideal case to be simple. In those conditions, the sample would mostly shrink with two different compression factors, a larger one $\gamma _Z$ along the normal axis and a smaller one $\gamma _\perp$ within the specimen plane (if $\gamma _\perp \simeq \gamma _X \simeq \gamma _Y$). This can be mathematically summarized by modeling the projection map (5) with the following:

6 $$\begin{aligned} \mathbf{x} = \Pi _\mathbf{e_Z} \circ \mathcal{R}_\mathbf{{u},\theta } \circ \mathcal{R}_{\mathbf{e_Z},\phi } \circ \mathcal{W}_{\omega } (\mathbf X), \end{aligned}$$

The linear part of the affine transformation $\mathcal{W}_{\omega }$ which describes the sample shrinkage is

7 $$\begin{aligned} \left[ \begin{array}{ccc} \gamma _{X} &{} 0 &{} 0 \\ 0 &{} \gamma _{Y} &{} 0 \\ 0 &{} 0 &{} \gamma _{Z} \\ \end{array} \right] . \end{aligned}$$

Here, the warping transformation $\mathcal{W}_{\omega }$ is indexed by the sample orientation ${\omega }=(\theta ,\phi )$, but $\mathcal{W}_{\omega }$ really depends on the accumulated dose and the micrograph index of the acquisition order. As the micrographs are collected, the sample will shrink, we assume exponentially, towards a stable plateau whose value is mostly a function of the electron dose rate [8].

8 $$\begin{aligned} \left\{ \begin{array}{rcl} \gamma _X &{} = &{} \gamma _{X,\infty } + (1-\gamma _{X,\infty }) e^{-i_\omega /\tau _X} \\ \gamma _Y&{} = &{} \gamma _{Y,\infty } + (1-\gamma _{Y,\infty }) e^{-i_\omega /\tau _Z} \\ \gamma _Z &{} = &{} \gamma _{Z,\infty } + (1-\gamma _{Z,\infty }) e^{-i_\omega /\tau _Z} \\ \end{array} \right. , \end{aligned}$$

here, $\gamma _{X,\infty }$, $\gamma _{Y,\infty }$ and $\gamma _{Z,\infty }$ are the plateau values for both shrinkage coefficients $\gamma _X$, $\gamma _Y$ and $\gamma _Z$; $i_\omega$ is the index of an electron micrograph; $\tau _X$, $\tau _Y$ and $\tau _Z$ are the associated shrinkage rates. Note that during the data acquisition with SerialEM [14], there is always a tracking and an autofocus routine happening between two recorded micrographs. Nevertheless, we can safely assume that $i_\omega$ is directly proportional to the accumulated dose.

A crude linear model with no coupling between axes for the sample deformation, as outlined by the Eqs. (6) , (7) and (8), can already capture some coarse variations of the projection map throughout the tilt series. It is very clear, when monitoring the quantity:

9 $$\begin{aligned} n_{3,\omega } = \Vert \mathbf{n_{1,\omega } } \times \mathbf{n_{2,\omega } } \Vert \end{aligned}$$

which becomes

10 $$\begin{aligned} n_{3,\omega } = \left[ ( \gamma _X^2 \cos ^2 \phi + \gamma _Y^2 \sin ^2 \phi ) \gamma _Z^2 \sin ^2 \theta + \gamma _X^2 \gamma _Y^2 \cos ^2 \theta \right] ^{1/2} \end{aligned}$$

for the deformation model defined in Eq. (7). In this case, if there was no sample warping ($\gamma _X=1$, $\gamma _Y=1$ and $\gamma _Z=1$), the quantity $n_{3,\omega }$ should remain equal to 1. On the other hand, with more shrinkage along the normal axis of the section, that is $1-\gamma _X,1-\gamma _Y \ll 1-\gamma _Z$, $n_{3,\omega }$ would oscillate between two curves specified by ${\gamma ^-_{\perp }}^2 \left[ 3 \gamma _Z^2 + {\gamma ^+_{\perp }}^2 \right] ^{1/2}/2$ and $\gamma ^-_{\perp } \gamma ^+_{\perp }$, with $\gamma ^-_{\perp } = \text{min}(\gamma _X,\gamma _Y)$ and $\gamma ^+_{\perp } = \text{max}(\gamma _X,\gamma _Y)$ during the multiple axis scheme with a tilt variation between −60° and +60°.

## Appendix 3: Weighted iterative approach

In this appendix, we elaborate the principles behind the weighted iterative approach.

Considering electrons traveling across a specimen, their trajectories $\gamma _{\mathbf{x},\omega }(t)$ depend on both their final destination $\mathbf{x}$ on the detector and the specimen orientation $\omega$; *t* is a variable that parametrizes a particle position along its path; $t=t_0$ and $t=t_1$ correspond to the specimen boundaries. The fundamental principle of electron tomography consists in inverting the following relation:

11 $$\begin{aligned} v(\mathbf{x},\omega ) = \mathcal{R}_\Gamma u(\mathbf{x},\omega ) \equiv \int\limits _{t_0}^{t_1} u[\gamma _{\mathbf{x},\omega }(t)] dt, \end{aligned}$$

which defines the Radon transform $\mathcal{R}_\Gamma$, $\Gamma$ being the set of all the possible electron trajectories; *u* is the volumic representation of the specimen; *v* is the projection micrograph density.

The formulation of the weighted iterative approach employed in this work involves the following operator $\bar{\mathcal{R}}^*_\Gamma$:

12 $$\begin{aligned} \bar{\mathcal{R}}^*_\Gamma v (\mathbf{X}) = \int\limits _{P_\omega (\mathbf{X})=\mathbf{x}} \lambda _{\mathbf{x},\omega }(\mathbf{X}) v(\mathbf{x},\omega ) \text{d}\omega , \end{aligned}$$

$\mathbf{X}$ is a variable representing the 3D positions in the specimen while $P_\omega$ are the projection maps associated with trajectories $\gamma _{\mathbf{x},\omega }(t)$; for every $(\mathbf{x},\omega ,t)$, we have $P_\omega (\gamma _{\mathbf{x},\omega }(t))=\mathbf{x}$; finally, $\lambda _\mathbf{x,\omega }$ is a function that modulates the back-projected densities depending on the position within the specimen and the source of the contribution, that is the originating micrograph.

In electron tomography, it is generally not possible to obtain the exact solution of the inverse problem outlined in Eq. (11), mostly because of the missing wedge problem. The mathematical system is *ill-posed*, and as a result, many artifacts hamper the reconstructions obtained via standard approaches.

The motivation behind introducing the weighting parameter in Eq. (12) is to enforce constraints on the back-projection operator that will ultimately lead to reconstruction bearing minimal flaw, but still be solution of Eq. (11). Possible choices for $\lambda _\mathbf{x,\omega }$ can be dictated by physics-based arguments. For example, to reduce the streaks effects surrounding dense objects, we can choose an expression that will effectively inflate their absorbance and attenuate the bleaching effect along the rays. Such an expression can be inferred using cross-validation ideas, with for instance,

13 $$\begin{aligned} \lambda _\mathbf{x,\omega } \sim - \int\limits _{P_\omega (X)=x,\omega \ne \omega '} v(x,\omega ') d \omega '. \end{aligned}$$

In addition, the original projection constraint along the rays must still be satisfied with:

14 $$\begin{aligned} \int\limits _{t_0}^{t_1} \lambda _\mathbf{x,\omega } \left( \gamma _\mathbf{x,\omega }(t)\right) dt =1, \end{aligned}$$

for every micrograph pixel $\mathbf{x}$ and every sample orientation $\omega$.

We have used for this work a SIRT-type iterative approach to find a solution for Eq. (11); the weighted-backprojection operator $\bar{\mathcal{R}}^*_\Gamma$ being used to correct for the discrepancies at each iteration. During the *i*th step, all the projections $v_i$ that correspond to the original specimen orientations are evaluated from the volume density $u_i$ through Eq. (11). Next, the volume density at iteration $i+1$ is evaluated by using

15 $$\begin{aligned} u_{i+1} = u_i + \alpha \bar{\mathcal{R}}^*_{\Gamma ,i} (v - v_i), \end{aligned}$$

with taking the following expression for the weighting operator in $\bar{\mathcal{R}}^*_{\Gamma ,i}$:

16 $$\begin{aligned} \lambda _{\mathbf{x},\omega ; i} (\mathbf{X}) = \frac{2}{\Delta t}- \frac{u_i(\mathbf{X})}{v_i(\mathbf{x},\omega )}, \end{aligned}$$

with $\Delta t = t_1-t_0$; $\alpha$ is just a mixing parameter for adjusting the convergence rate.

Equation (16) approximates Eq. (13) and satisfies relation (14). Expressed in this way, the system of Eqs. (15), (16) is similar to the adaptive approach developed in [22].

The iterative process is initialized by simply back-projecting the raw electron micrographs.

Finally, we note that the weighted iterative approach described in this appendix is tightly related to the multiplicative-type algorithm category in image reconstruction; for instance, the expectation maximization algorithm in emission tomography (see chap. 5 in  [24]). For this latter, reconstructions are obtained by maximizing a likelihood function characterizing the physical nature of the phenomenon, a Poisson distribution. The procedure is implemented through a multiplicative iterative scheme, and can be shown equivalent to Eq. (15) with $\alpha =1$. This statistical framework is also very appropriate in the case of electron tomography and its underlying physical principles. It also offers, as discussed above in the context of cross-validation, a more refined solution to artifacts compared to more standard approaches (FBP, SIRT, etc.).

## References

[1] Shu, X., Lev-Ram, V., Deerinck, T.J., Qi, Y., Ramko, E.B., Davidson, M.W., Jin, Y., Ellisman, M.H., Tsien, R.Y.: A genetically encoded tag for correlated light and electron microscopy of intact cells, tissues, and organisms. PLoS Biol. **9**(4), e1001041 (2011) 21483721 10.1371/journal.pbio.1001041 PMC3071375

[2] Martell, J.D., Deerinck, T.J., Sancak, Y., Poulos, T.L., Mootha, V.K., Sosinsky, G.E., Ellisman, M.H., Ting, A.Y.: Engineered ascorbate peroxidase as a genetically encoded reporter for electron microscopy. Nat. Biotechnol. **30**, 1143–1148 (2012) 10.1038/nbt.2375 PMC3699407 23086203

[3] Ngo, J.T., Adams, S.R., Deerinck, T.J., Boassa, D., Rodriguez-Rivera, F., Palida, S.F., Bertozzi, C.R., Ellisman, M.H., Tsien, R.Y.: Click-EM for imaging metabolically tagged non-protein biomolecules. Nat. Chem. Biol. **12**(6), 459–465 (2016). doi:10.1038/nchembio.2076 27110681 10.1038/nchembio.2076 PMC4871776

[4] Frank, J.: Electron Tomography: Methods for Three-Dimensional Visualization of Structure in the Cell. Springer, New York (2006)

[5] McEwen, B.F., Renken, C., Marko, M., Mannella, C.: Principles and Practice in Electron Tomography, pp. 129–168. Academic Press, San Diego (2008) 10.1016/S0091-679X(08)00606-7 19118675

[6] Hawkes, P.W., Kasper, E.: Principles of Electron Optics. Academic Press Inc, San Diego (1989)

[7] Reimer, L., Kohl, H.: Transmission Electron Microscopy: Physics of Image Formation. Springer Verlag, Berlin (2008)

[8] Luther, P.K.: Sample shrinkage and radiation damage of plastic sections. In: Frank, J. (ed.) Electron Tomography: Methods for Three-Dimensional Visualization of Structure in the Cell, pp. 17–40. Springer, Berlin (2006)

[9] Mastronarde, D.N.: Fiducial marker and hybrid alignment methods for single- and double-axis tomography. In: Frank, J. (ed.) Electron Tomography: Methods for Three-Dimensional Visualization of Structure in the Cell, pp. 163–185. Springer, Berlin (2006)

[10] Cantele, F., Paccagnini, E., Pigino, G., Lupetti, P., Lanzavecchia, S.: Simultaneous alignment of dual-axis tilt series. J. Struc. Biol. **169**(2), 192–199 (2010) 10.1016/j.jsb.2009.10.003 19818858

[11] Xiao, C., Kuznetsov, Y.G., Sun, S., Hafenstein, S.L., Kostyuchenko, V.A., Chipman, P.R., Suzan-Monti, M., Raoult, D., McPherson, A., Rossmann, M.G.: Structural studies of the giant Mimivirus. PLoS. Biol. **7**(4), e1000092 (2009) 19402750 10.1371/journal.pbio.1000092 PMC2671561

[12] Klose, T., Kuznetsov, Y.G., Xiao, C., Sun, S., McPherson, A., Rossmann, M.G.: The three-dimensional structure of Mimivirus. Intervirology **53**(5), 268–273 (2010) 20551678 10.1159/000312911 PMC2895761

[13] Mutsafi, Y., Shimoni, E., Shimon, A., Minsky, A.: Membrane assembly during the infection cycle of the giant Mimivirus. PLoS Pathog. **9**(5), e1003367 (2013) 23737745 10.1371/journal.ppat.1003367 PMC3667779

[14] Mastronarde, D.N.: Automated electron microscope tomography using robust prediction of specimen movements. J. Struct. Biol. **152**(1), 36–51 (2005) 16182563 10.1016/j.jsb.2005.07.007

[15] Guan, H., Gordon, R.: A projection access order for speedy convergence of art (algebraic reconstruction technique): a multilevel scheme for computed tomography. Phys. Med. Biol. **39**(11), 2005–2022 (1994) 15560007 10.1088/0031-9155/39/11/013

[16] Lawrence, A.F., Bouwer, J.C., Perkins, G., Ellisman, M.H.: Transform-based backprojection for volume reconstruction of large format electron microscope tilt series. J. Struct. Biol. **154**, 144–167 (2006) 16542854 10.1016/j.jsb.2005.12.012

[17] Phan, S., Bouwer, J., Lanman, J., Terada, M., Lawrence, A.F.: Non-linear bundle adjustment for electron tomography, In: Proceedings of the 2009 WRI World Congress on Computer Science and Information Engineering-Volume 01, CSIE ’09, IEEE Computer Society, Washington, 2009, pp. 604–612

[18] Phan, S., Lawrence, A., Molina, T., Lanman, J., Berlanga, M., Terada, M., Kulungowski, A., Obayashi, J., Ellisman, M.: Txbr montage reconstruction for large field electron tomography. J. Struct. Biol. **180**(1), 154–164 (2012) 22749959 10.1016/j.jsb.2012.06.006

[19] Mastronarde, D.N.: Dual-axis tomography: an approach with alignment methods that preserve resolution. J. Struct. Biol. **120**(3), 343–352 (1997) 9441937 10.1006/jsbi.1997.3919

[20] Mastronarde, D.N.: Tomographic reconstruction with the imod software package. Microsc. Microanal. **12**(2), 178–179 (2006)17481355

[21] Cao, M., Takaoka, A., Zhang, H.B., Nishi, R.: An automatic method of detecting and tracking fiducial markers for alignment in electron tomography. J. Electron. Microsc. (Tokyo) **60**(1), 39–46 (2011) 21075783 10.1093/jmicro/dfq076

[22] Wan, X., Zhang, F., Chu, Q., Zhang, K., Sun, F., Yuan, B., Liu, Z.: Three-dimensional reconstruction using an adaptive simultaneous algebraic reconstruction technique in electron tomography. J. Struct. Biol. **175**(3), 277–287 (2011) 21699984 10.1016/j.jsb.2011.06.002

[23] Wan, X., Phan, S., Lawrence, A., Zhang, F., Han, R., Liu, Z., Ellisman, M.: Iterative methods in large field electron microscope tomography. SIAM. J. Sci. Comput. **35**(5), S402–S419 (2013). doi:10.1137/120881464

[24] Natterer, F., Wübbeling, F.: Mathematical methods in image reconstruction. Society for Industrial and Applied Mathematics (2001). http://epubs.siam.org/doi/abs/10.1137/1.9780898718324

[25] Bracewell, R.N.: R. A. C., Inversion of a fan-beam scans in radio astronomy. Astrophys. J. **150**, 427–434 (1967)

[26] Saxton, W., Baumeister, W., Hahn, M.: Three-dimensional reconstruction of imperfect two-dimensional crystals. Ultramicroscopy **13**, 57–70 (1984) 6382732 10.1016/0304-3991(84)90057-3

[27] Penczek, P.A., Frank, J.: Resolution in electron tomography. In: Frank, J. (ed.) Electron Tomography: Methods for Three-Dimensional Visualization of Structure in the Cell, pp. 307–330. Springer, Berlin (2006)

[28] Raoult, D., Scola, B.L., Birtles, R.: The discovery and characterization of Mimivirus, the largest known virus and putative pneumonia agent. Clin. Infect. Dis. **45**(1), 95–102 (2007) 17554709 10.1086/518608

[29] Fotin, A., Cheng, Y., Sliz, P., Grigorieff, N., Harrison, S.C., Kirchhausen, T., Walz, T.: Molecular model for a complete clathrin lattice from electron cryomicroscopy. Nature **432**(7017), 573–579 (2004) 15502812 10.1038/nature03079

[30] Cheng, Y., Boll, W., Kirchhausen, T., Harrison, S.C., Walz, T.: Cryo-electron tomography of clathrin-coated vesicles: structural implications for coat assembly. J. Mol. Biol. **365**(3), 892–899 (2007) 17095010 10.1016/j.jmb.2006.10.036 PMC1839968

[31] Nagayama, K.: Another 60 years in electron microscopy: development of phase-plate electron microscopy and biological applications. J. Electr. Microsc. **60**(suppl 1), S43–S62 (2011) 10.1093/jmicro/dfr037 21844600

[32] Xiong, Q., Morphew, M.K., Schwartz, C.L., Hoenger, A.H., Mastronarde, D.N.: CTF determination and correction for low dose tomographic tilt series. J. Struct. Biol. **168**(3), 378–387 (2009) 19732834 10.1016/j.jsb.2009.08.016 PMC2784817

[33] Zhou, Z.H.: Towards atomic resolution structural determination by single-particle cryo-electron microscopy. Curr. Opin. Struct. Biol. **18**(2), 218–228 (2008) 18403197 10.1016/j.sbi.2008.03.004 PMC2714865

[34] Briggs, J.A.: Structural biology in situ—potential of subtomogram averaging. Curr. Opin. Struc. Biol. **23**(2), 261–267 (2013). Theory and simulation/macromolecular assemblies 10.1016/j.sbi.2013.02.003 23466038

[35] Nicastro, D., Schwartz, C., Pierson, J., Gaudette, R., Porter, M.E., McIntosh, J.R.: The molecular architecture of axonemes revealed by cryoelectron tomography. Science **313**(5789), 944–948 (2006) 16917055 10.1126/science.1128618

[36] Jones, E., Oliphant, T., Peterson, P., et al.: SciPy: open source scientific tools for python (2001). http://www.scipy.org/

